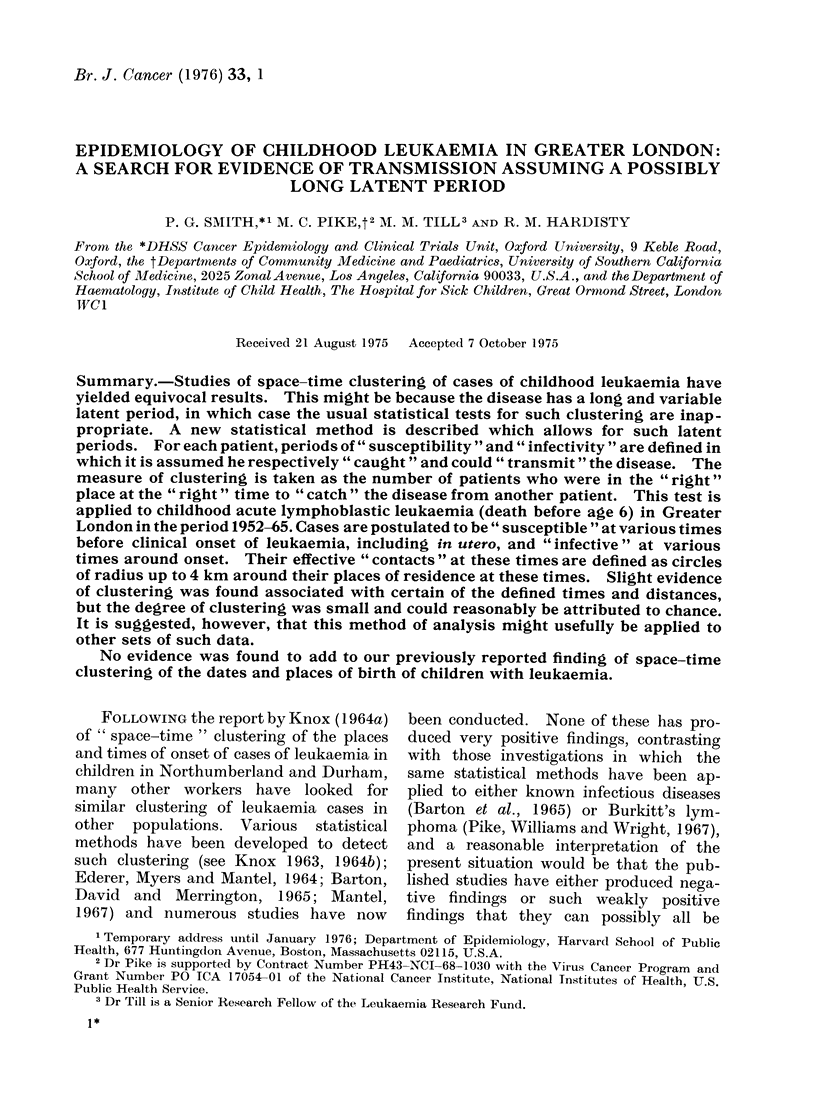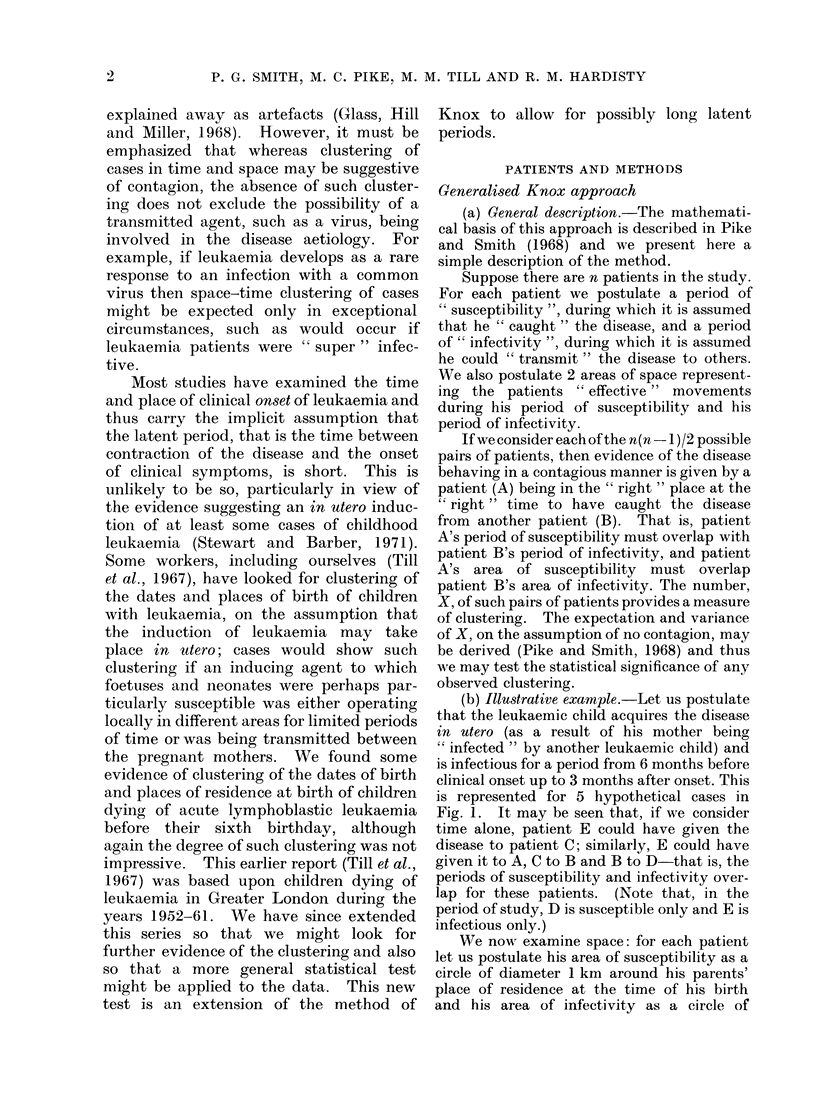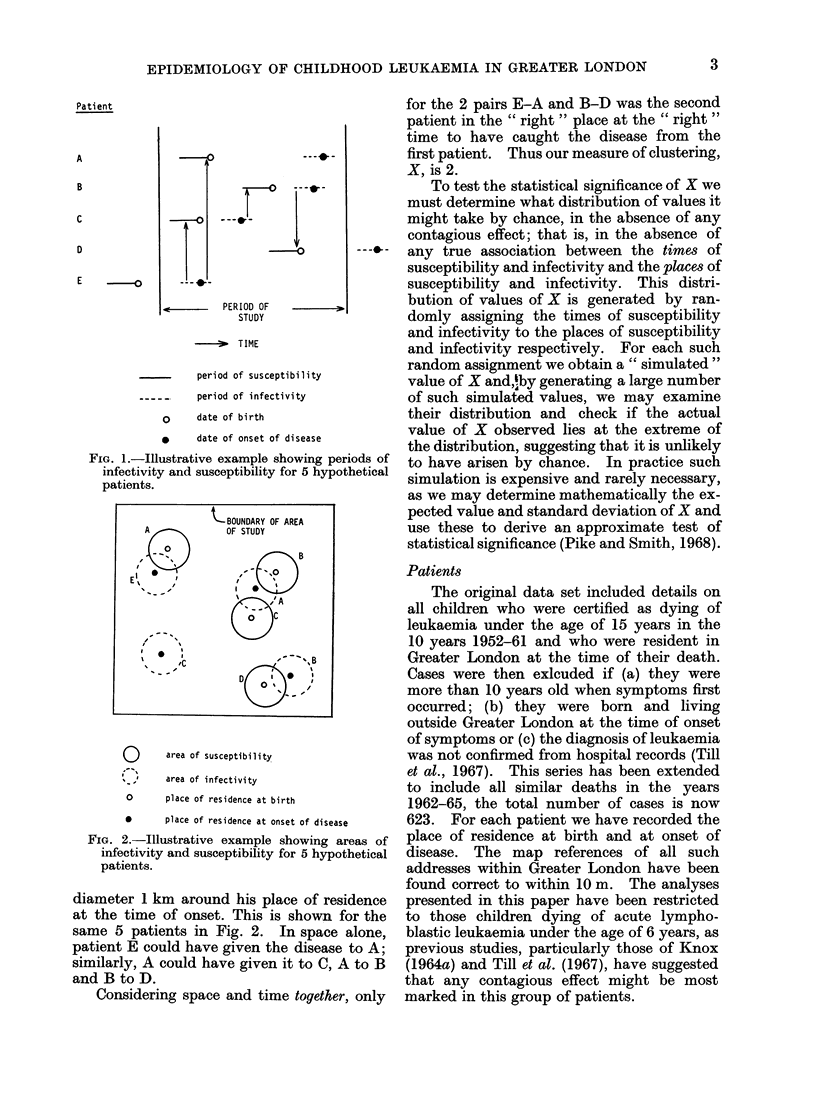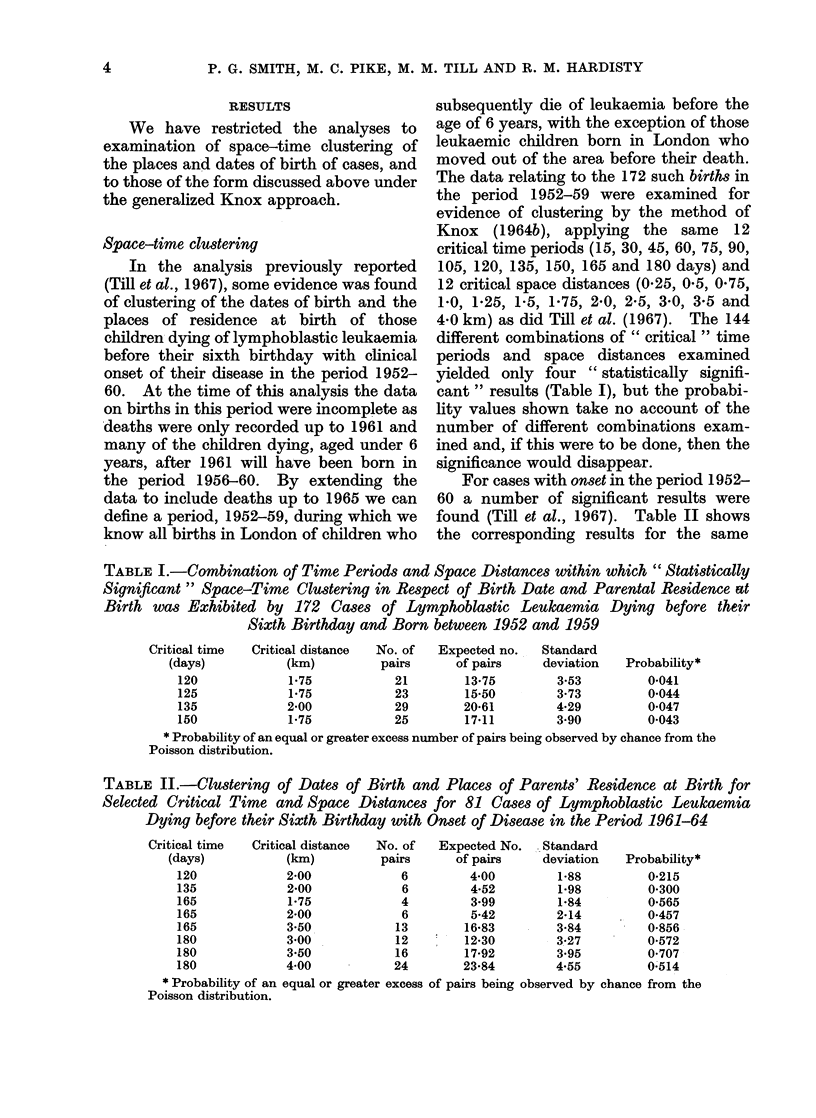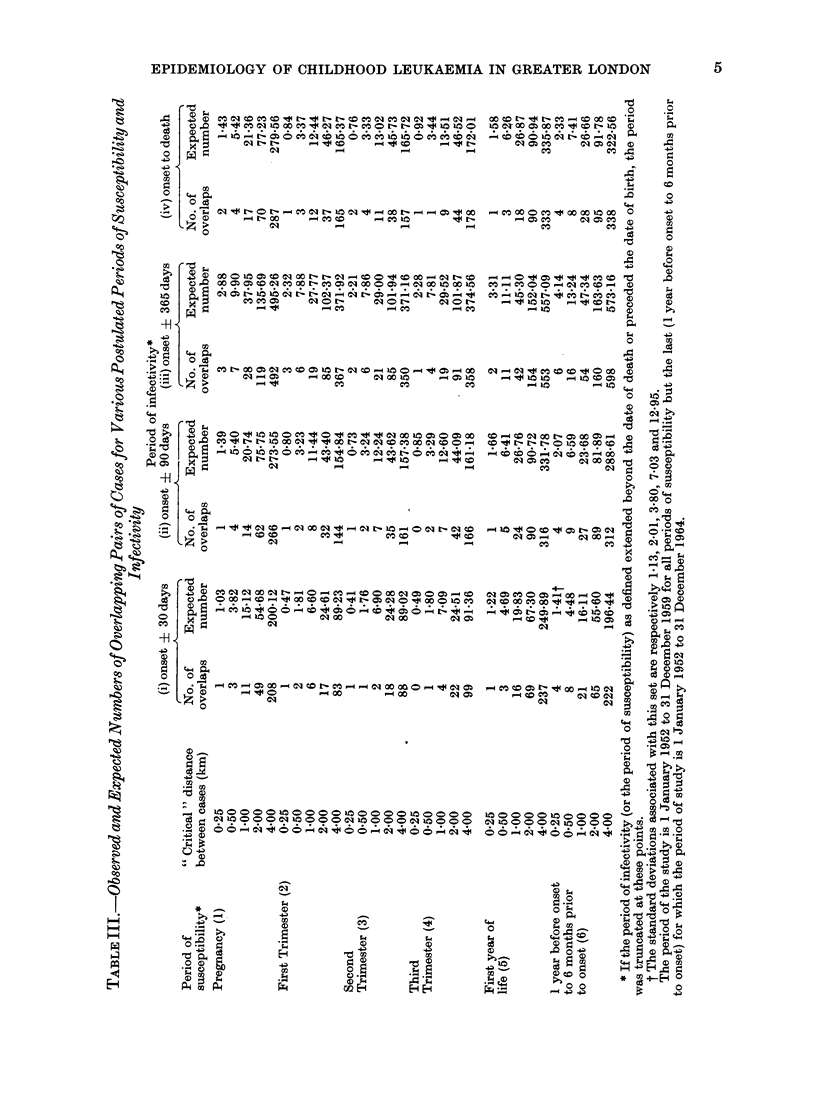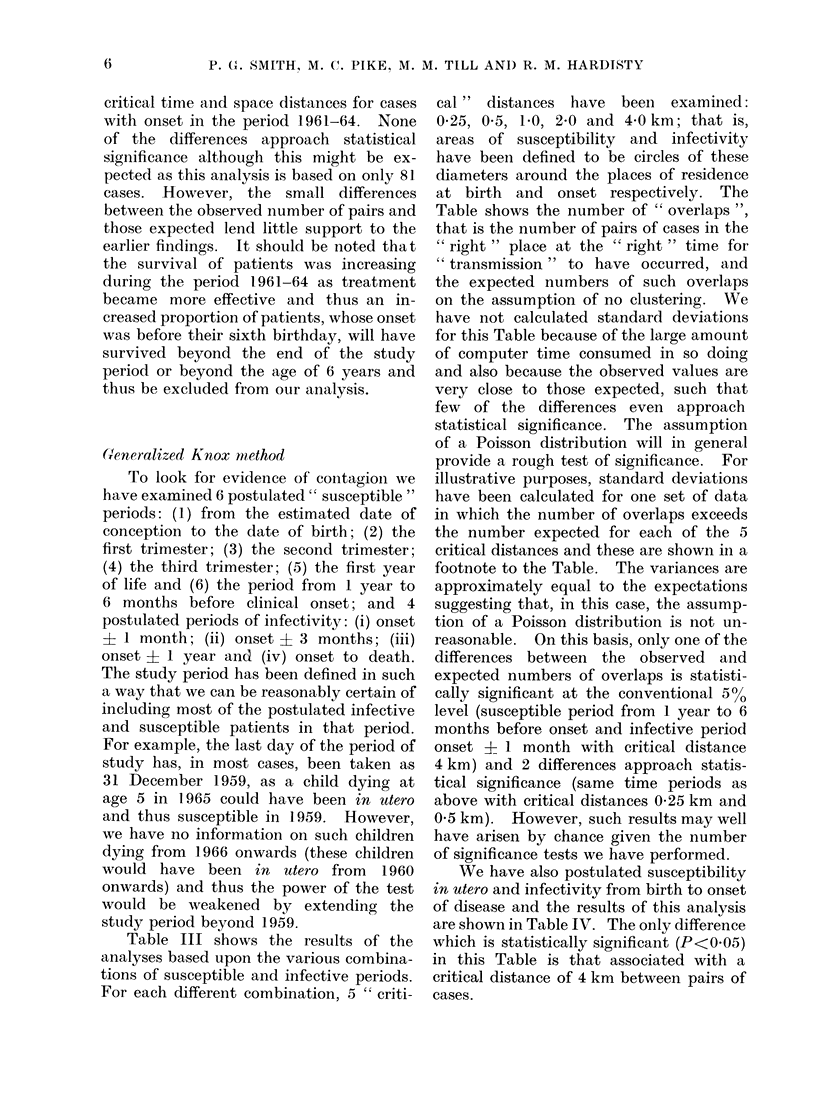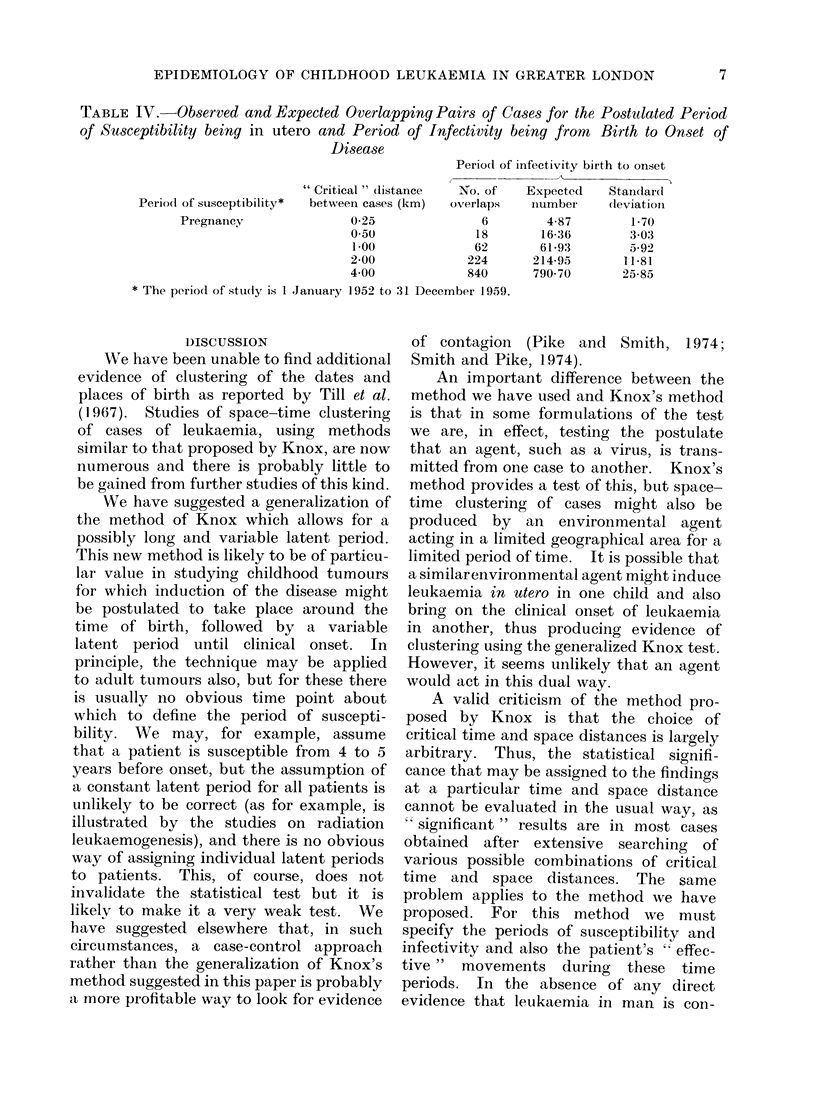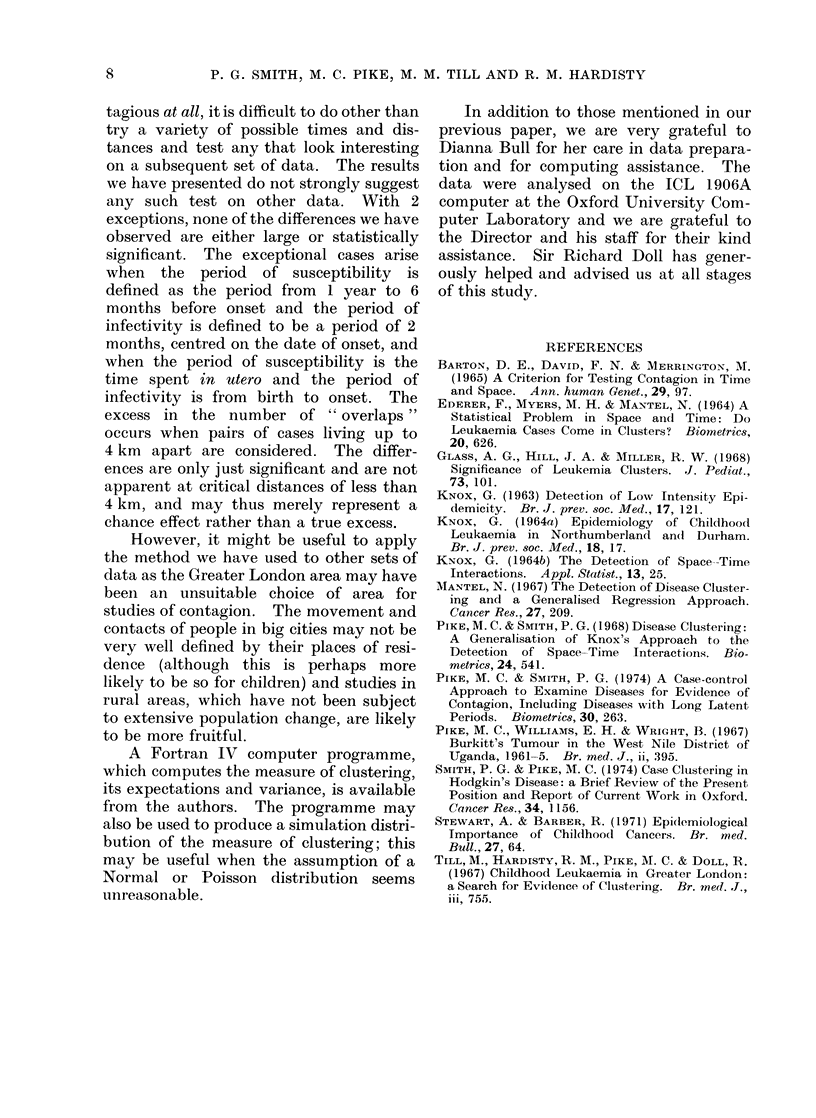# Epidemiology of childhood leukaemia in greater london: A search for evidence of transmission assuming a possibly long latent period.

**DOI:** 10.1038/bjc.1976.1

**Published:** 1976-01

**Authors:** P. G. Smith, M. C. Pike, M. M. Till, R. M. Hardisty

## Abstract

Studies of space-time clustering of cases of childhood leukaemia have yielded equivocal results. This might be because the disease has a long and variable latent period, in which case the usual statistical tests for such clustering are inappropriate. A new statistical method is described which allows for such latent periods. For each patient, periods of "susceptibility" and "infectivity" are defined in which it is assumed he respectively "caught" and could "transmit" the disease. The measure of clustering is taken as the number of patients who were in the "right" place at the "right" time to "catch" the disease from another patient. This test is applied to childhood acute lymphoblastic leukaemia (death before age 6) in Greater London in the period 1952-65. Cases are postulated to be "susceptible" at various times before clinical onset of leukaemia, including in utero, and "infective" at various times around onset. Their effective "contacts" at these times are defined as circles of radius up to 4 km around their places of residence at these times. Slight evidence of clustering was found associated with certain of the defined times and distances, but the degree of clustering was small and could reasonably be attributed to chance. It is suggested, however, that this method of analysis might usefully be applied to other sets of such data. No evidence was found to add to our previously reported finding of space-time clustering of the dates and places of birth of children with leukaemia.


*Br. J. Cancer* (1976) **33**, 1

### EPIDEMIOLOGY OF CHILDHOOD LEUKAEMIA IN GREATER LONDON: A SEARCH FOR EVIDENCE OF TRANSMISSION ASSUMING A POSSIBLY LONG LATENT PERIOD

P. G. SMITH,*[1] M. C. PIKE,†[2] M. M. TILL[3] AND R. M. HARDISTY

*From the *DHSS Cancer Epidemiology and Clinical Trials Unit, Oxford University, 9 Keble Road, Oxford, the †Departments of Community Medicine and Paediatrics, University of Southern California School of Medicine, 2025 Zonal Avenue, Los Angeles, California 90033, U.S.A., and the Department of Haematology, Institute of Child Health, The Hospital for Sick Children, Great Ormond Street, London WC1*

Received 21 August 1975 Accepted 7 October 1975

**Summary.—Studies of space–time clustering of cases of childhood leukaemia have yielded equivocal results. This might be because the disease has a long and variable latent period, in which case the usual statistical tests for such clustering are inappropriate. A new statistical method is described which allows for such latent periods. For each patient, periods of "susceptibility" and "infectivity" are defined in which it is assumed he respectively "caught" and could "transmit" the disease. The measure of clustering is taken as the number of patients who were in the "right" place at the "right" time to "catch" the disease from another patient. This test is applied to childhood acute lymphoblastic leukaemia (death before age 6) in Greater London in the period 1952–65. Cases are postulated to be "susceptible" at various times before clinical onset of leukaemia, including *in utero*, and "infective" at various times around onset. Their effective "contacts" at these times are defined as circles of radius up to 4 km around their places of residence at these times. Slight evidence of clustering was found associated with certain of the defined times and distances, but the degree of clustering was small and could reasonably be attributed to chance. It is suggested, however, that this method of analysis might usefully be applied to other sets of such data.**

**No evidence was found to add to our previously reported finding of space–time clustering of the dates and places of birth of children with leukaemia.**

FOLLOWING the report by Knox (1964*a*) of "space–time" clustering of the places and times of onset of cases of leukaemia in children in Northumberland and Durham, many other workers have looked for similar clustering of leukaemia cases in other populations. Various statistical methods have been developed to detect such clustering (see Knox 1963, 1964*b*); Ederer, Myers and Mantel, 1964; Barton, David and Merrington, 1965; Mantel, 1967) and numerous studies have now been conducted. None of these has produced very positive findings, contrasting with those investigations in which the same statistical methods have been applied to either known infectious diseases (Barton *et al.*, 1965) or Burkitt's lymphoma (Pike, Williams and Wright, 1967), and a reasonable interpretation of the present situation would be that the published studies have either produced negative findings or such weakly positive findings that they can possibly all be

[1] Temporary address until January 1976; Department of Epidemiology, Harvard School of Public Health, 677 Huntingdon Avenue, Boston, Massachusetts 02115, U.S.A.

[2] Dr Pike is supported by Contract Number PH43–NCI–68–1030 with the Virus Cancer Program and Grant Number PO ICA 17054–01 of the National Cancer Institute, National Institutes of Health, U.S. Public Health Service.

[3] Dr Till is a Senior Research Fellow of the Leukaemia Research Fund.

explained away as artefacts (Glass, Hill and Miller, 1968). However, it must be emphasized that whereas clustering of cases in time and space may be suggestive of contagion, the absence of such clustering does not exclude the possibility of a transmitted agent, such as a virus, being involved in the disease aetiology. For example, if leukaemia develops as a rare response to an infection with a common virus then space–time clustering of cases might be expected only in exceptional circumstances, such as would occur if leukaemia patients were " super " infective.

Most studies have examined the time and place of clinical *onset* of leukaemia and thus carry the implicit assumption that the latent period, that is the time between contraction of the disease and the onset of clinical symptoms, is short. This is unlikely to be so, particularly in view of the evidence suggesting an *in utero* induction of at least some cases of childhood leukaemia (Stewart and Barber, 1971). Some workers, including ourselves (Till *et al.*, 1967), have looked for clustering of the dates and places of birth of children with leukaemia, on the assumption that the induction of leukaemia may take place *in utero*; cases would show such clustering if an inducing agent to which foetuses and neonates were perhaps particularly susceptible was either operating locally in different areas for limited periods of time or was being transmitted between the pregnant mothers. We found some evidence of clustering of the dates of birth and places of residence at birth of children dying of acute lymphoblastic leukaemia before their sixth birthday, although again the degree of such clustering was not impressive. This earlier report (Till *et al.*, 1967) was based upon children dying of leukaemia in Greater London during the years 1952–61. We have since extended this series so that we might look for further evidence of the clustering and also so that a more general statistical test might be applied to the data. This new test is an extension of the method of Knox to allow for possibly long latent periods.

### PATIENTS AND METHODS

### *Generalised Knox approach*

(a) *General description.*—The mathematical basis of this approach is described in Pike and Smith (1968) and we present here a simple description of the method.

Suppose there are $n$ patients in the study. For each patient we postulate a period of " susceptibility ", during which it is assumed that he " caught " the disease, and a period of " infectivity ", during which it is assumed he could " transmit " the disease to others. We also postulate 2 areas of space representing the patients " effective " movements during his period of susceptibility and his period of infectivity.

If we consider each of the $n(n-1)/2$ possible pairs of patients, then evidence of the disease behaving in a contagious manner is given by a patient (A) being in the " right " place at the " right " time to have caught the disease from another patient (B). That is, patient A's period of susceptibility must overlap with patient B's period of infectivity, and patient A's area of susceptibility must overlap patient B's area of infectivity. The number, $X$, of such pairs of patients provides a measure of clustering. The expectation and variance of $X$, on the assumption of no contagion, may be derived (Pike and Smith, 1968) and thus we may test the statistical significance of any observed clustering.

(b) *Illustrative example.*—Let us postulate that the leukaemic child acquires the disease *in utero* (as a result of his mother being " infected " by another leukaemic child) and is infectious for a period from 6 months before clinical onset up to 3 months after onset. This is represented for 5 hypothetical cases in Fig. 1. It may be seen that, if we consider time alone, patient E could have given the disease to patient C; similarly, E could have given it to A, C to B and B to D—that is, the periods of susceptibility and infectivity overlap for these patients. (Note that, in the period of study, D is susceptible only and E is infectious only.)

We now examine space: for each patient let us postulate his area of susceptibility as a circle of diameter 1 km around his parents' place of residence at the time of his birth and his area of infectivity as a circle of

FIG. 1.—Illustrative example showing periods of infectivity and susceptibility for 5 hypothetical patients.

FIG. 2.—Illustrative example showing areas of infectivity and susceptibility for 5 hypothetical patients.

diameter 1 km around his place of residence at the time of onset. This is shown for the same 5 patients in Fig. 2. In space alone, patient E could have given the disease to A; similarly, A could have given it to C, A to B and B to D.

Considering space and time *together*, only for the 2 pairs E–A and B–D was the second patient in the " right " place at the " right " time to have caught the disease from the first patient. Thus our measure of clustering, $X$, is 2.

To test the statistical significance of $X$ we must determine what distribution of values it might take by chance, in the absence of any contagious effect; that is, in the absence of any true association between the *times* of susceptibility and infectivity and the *places* of susceptibility and infectivity. This distribution of values of $X$ is generated by randomly assigning the times of susceptibility and infectivity to the places of susceptibility and infectivity respectively. For each such random assignment we obtain a " simulated " value of $X$ and, by generating a large number of such simulated values, we may examine their distribution and check if the actual value of $X$ observed lies at the extreme of the distribution, suggesting that it is unlikely to have arisen by chance. In practice such simulation is expensive and rarely necessary, as we may determine mathematically the expected value and standard deviation of $X$ and use these to derive an approximate test of statistical significance (Pike and Smith, 1968).

*Patients*

The original data set included details on all children who were certified as dying of leukaemia under the age of 15 years in the 10 years 1952–61 and who were resident in Greater London at the time of their death. Cases were then exlcuded if (a) they were more than 10 years old when symptoms first occurred; (b) they were born and living outside Greater London at the time of onset of symptoms or (c) the diagnosis of leukaemia was not confirmed from hospital records (Till *et al.*, 1967). This series has been extended to include all similar deaths in the years 1962–65, the total number of cases is now 623. For each patient we have recorded the place of residence at birth and at onset of disease. The map references of all such addresses within Greater London have been found correct to within 10 m. The analyses presented in this paper have been restricted to those children dying of acute lymphoblastic leukaemia under the age of 6 years, as previous studies, particularly those of Knox (1964*a*) and Till *et al.* (1967), have suggested that any contagious effect might be most marked in this group of patients.

### RESULTS

We have restricted the analyses to examination of space–time clustering of the places and dates of birth of cases, and to those of the form discussed above under the generalized Knox approach.

### *Space–time clustering*

In the analysis previously reported (Till *et al.*, 1967), some evidence was found of clustering of the dates of birth and the places of residence at birth of those children dying of lymphoblastic leukaemia before their sixth birthday with clinical onset of their disease in the period 1952–60. At the time of this analysis the data on births in this period were incomplete as deaths were only recorded up to 1961 and many of the children dying, aged under 6 years, after 1961 will have been born in the period 1956–60. By extending the data to include deaths up to 1965 we can define a period, 1952–59, during which we know all births in London of children who subsequently die of leukaemia before the age of 6 years, with the exception of those leukaemic children born in London who moved out of the area before their death. The data relating to the 172 such *births* in the period 1952–59 were examined for evidence of clustering by the method of Knox (1964*b*), applying the same 12 critical time periods (15, 30, 45, 60, 75, 90, 105, 120, 135, 150, 165 and 180 days) and 12 critical space distances (0·25, 0·5, 0·75, 1·0, 1·25, 1·5, 1·75, 2·0, 2·5, 3·0, 3·5 and 4·0 km) as did Till *et al.* (1967). The 144 different combinations of " critical " time periods and space distances examined yielded only four " statistically significant " results (Table I), but the probability values shown take no account of the number of different combinations examined and, if this were to be done, then the significance would disappear.

For cases with *onset* in the period 1952–60 a number of significant results were found (Till *et al.*, 1967). Table II shows the corresponding results for the same

TABLE I.—*Combination of Time Periods and Space Distances within which " Statistically Significant " Space–Time Clustering in Respect of Birth Date and Parental Residence at Birth was Exhibited by 172 Cases of Lymphoblastic Leukaemia Dying before their Sixth Birthday and Born between 1952 and 1959*

| Critical time (days) | Critical distance (km) | No. of pairs | Expected no. of pairs | Standard deviation | Probability* |
|---|---|---|---|---|---|
| 120 | 1·75 | 21 | 13·75 | 3·53 | 0·041 |
| 125 | 1·75 | 23 | 15·50 | 3·73 | 0·044 |
| 135 | 2·00 | 29 | 20·61 | 4·29 | 0·047 |
| 150 | 1·75 | 25 | 17·11 | 3·90 | 0·043 |

* Probability of an equal or greater excess number of pairs being observed by chance from the Poisson distribution.

TABLE II.—*Clustering of Dates of Birth and Places of Parents' Residence at Birth for Selected Critical Time and Space Distances for 81 Cases of Lymphoblastic Leukaemia Dying before their Sixth Birthday with Onset of Disease in the Period 1961–64*

| Critical time (days) | Critical distance (km) | No. of pairs | Expected No. of pairs | Standard deviation | Probability* |
|---|---|---|---|---|---|
| 120 | 2·00 | 6 | 4·00 | 1·88 | 0·215 |
| 135 | 2·00 | 6 | 4·52 | 1·98 | 0·300 |
| 165 | 1·75 | 4 | 3·99 | 1·84 | 0·565 |
| 165 | 2·00 | 6 | 5·42 | 2·14 | 0·457 |
| 165 | 3·50 | 13 | 16·83 | 3·84 | 0·856 |
| 180 | 3·00 | 12 | 12·30 | 3·27 | 0·572 |
| 180 | 3·50 | 16 | 17·92 | 3·95 | 0·707 |
| 180 | 4·00 | 24 | 23·84 | 4·55 | 0·514 |

* Probability of an equal or greater excess of pairs being observed by chance from the Poisson distribution.

TABLE III.—*Observed and Expected Numbers of Overlapping Pairs of Cases for Various Postulated Periods of Susceptibility and Infectivity*

| | | Period of infectivity* | | | | | | | |
|---|---|---|---|---|---|---|---|---|---|
| | | (i) onset ± 30 days | | (ii) onset ± 90 days | | (iii) onset ± 365 days | | (iv) onset to death | |
| Period of susceptibility* | "Critical" distance between cases (km) | No. of overlaps | Expected number | No. of overlaps | Expected number | No. of overlaps | Expected number | No. of overlaps | Expected number |
| Pregnancy (1) | 0·25 | 1 | 1·03 | 1 | 1·39 | 3 | 2·88 | 2 | 1·43 |
| | 0·50 | 3 | 3·82 | 4 | 5·40 | 7 | 9·90 | 4 | 5·42 |
| | 1·00 | 11 | 15·12 | 14 | 20·74 | 28 | 37·95 | 17 | 21·36 |
| | 2·00 | 49 | 54·68 | 62 | 75·75 | 119 | 135·69 | 70 | 77·23 |
| | 4·00 | 208 | 200·12 | 266 | 273·55 | 492 | 495·26 | 287 | 279·56 |
| First Trimester (2) | 0·25 | 1 | 0·47 | 1 | 0·80 | 3 | 2·32 | 1 | 0·84 |
| | 0·50 | 2 | 1·81 | 2 | 3·23 | 6 | 7·88 | 3 | 3·37 |
| | 1·00 | 6 | 6·60 | 8 | 11·44 | 19 | 27·77 | 12 | 12·44 |
| | 2·00 | 17 | 24·61 | 32 | 43·40 | 85 | 102·37 | 37 | 46·27 |
| | 4·00 | 83 | 89·23 | 144 | 154·84 | 367 | 371·92 | 165 | 165·37 |
| Second Trimester (3) | 0·25 | 1 | 0·41 | 1 | 0·73 | 2 | 2·21 | 2 | 0·76 |
| | 0·50 | 1 | 1·76 | 2 | 3·24 | 6 | 7·86 | 4 | 3·33 |
| | 1·00 | 2 | 6·90 | 7 | 12·24 | 21 | 29·00 | 11 | 13·02 |
| | 2·00 | 18 | 24·28 | 35 | 43·62 | 85 | 101·94 | 38 | 45·73 |
| | 4·00 | 88 | 89·02 | 161 | 157·38 | 350 | 371·16 | 157 | 165·72 |
| Third Trimester (4) | 0·25 | 0 | 0·49 | 0 | 0·85 | 1 | 2·28 | 1 | 0·92 |
| | 0·50 | 1 | 1·80 | 2 | 3·29 | 4 | 7·81 | 1 | 3·44 |
| | 1·00 | 4 | 7·09 | 7 | 12·60 | 19 | 29·52 | 9 | 13·51 |
| | 2·00 | 22 | 24·51 | 42 | 44·09 | 91 | 101·87 | 44 | 46·52 |
| | 4·00 | 99 | 91·36 | 166 | 161·18 | 358 | 374·56 | 178 | 172·01 |
| First year of life (5) | 0·25 | 1 | 1·22 | 1 | 1·66 | 2 | 3·31 | 1 | 1·58 |
| | 0·50 | 3 | 4·69 | 5 | 6·41 | 11 | 11·11 | 3 | 6·26 |
| | 1·00 | 16 | 19·83 | 24 | 26·76 | 42 | 45·30 | 18 | 26·87 |
| | 2·00 | 69 | 67·30 | 90 | 90·72 | 154 | 152·04 | 90 | 90·94 |
| | 4·00 | 237 | 249·89 | 316 | 331·78 | 553 | 557·09 | 333 | 335·87 |
| 1 year before onset to 6 months prior to onset (6) | 0·25 | 4 | 1·41† | 4 | 2·07 | 6 | 4·14 | 4 | 2·33 |
| | 0·50 | 8 | 4·48 | 9 | 6·59 | 16 | 13·24 | 8 | 7·41 |
| | 1·00 | 21 | 16·11 | 27 | 23·68 | 54 | 47·34 | 28 | 26·66 |
| | 2·00 | 65 | 55·60 | 89 | 81·89 | 160 | 163·63 | 95 | 91·78 |
| | 4·00 | 222 | 196·44 | 312 | 288·61 | 598 | 573·16 | 338 | 322·56 |

* If the period of infectivity (or the period of susceptibility) as defined extended beyond the date of death or preceded the date of birth, the period was truncated at these points.

† The standard deviations associated with this set are respectively 1·13, 2·01, 3·80, 7·03 and 12·95.

The period of the study is 1 January 1952 to 31 December 1959 for all periods of susceptibility but the last (1 year before onset to 6 months prior to onset) for which the period of study is 1 January 1952 to 31 December 1964.

critical time and space distances for cases with onset in the period 1961–64. None of the differences approach statistical significance although this might be expected as this analysis is based on only 81 cases. However, the small differences between the observed number of pairs and those expected lend little support to the earlier findings. It should be noted that the survival of patients was increasing during the period 1961–64 as treatment became more effective and thus an increased proportion of patients, whose onset was before their sixth birthday, will have survived beyond the end of the study period or beyond the age of 6 years and thus be excluded from our analysis.

*Generalized Knox method*

To look for evidence of contagion we have examined 6 postulated " susceptible " periods: (1) from the estimated date of conception to the date of birth; (2) the first trimester; (3) the second trimester; (4) the third trimester; (5) the first year of life and (6) the period from 1 year to 6 months before clinical onset; and 4 postulated periods of infectivity: (i) onset $\pm$ 1 month; (ii) onset $\pm$ 3 months; (iii) onset $\pm$ 1 year and (iv) onset to death. The study period has been defined in such a way that we can be reasonably certain of including most of the postulated infective and susceptible patients in that period. For example, the last day of the period of study has, in most cases, been taken as 31 December 1959, as a child dying at age 5 in 1965 could have been *in utero* and thus susceptible in 1959. However, we have no information on such children dying from 1966 onwards (these children would have been *in utero* from 1960 onwards) and thus the power of the test would be weakened by extending the study period beyond 1959.

Table III shows the results of the analyses based upon the various combinations of susceptible and infective periods. For each different combination, 5 " critical " distances have been examined: 0·25, 0·5, 1·0, 2·0 and 4·0 km; that is, areas of susceptibility and infectivity have been defined to be circles of these diameters around the places of residence at birth and onset respectively. The Table shows the number of " overlaps ", that is the number of pairs of cases in the " right " place at the " right " time for " transmission " to have occurred, and the expected numbers of such overlaps on the assumption of no clustering. We have not calculated standard deviations for this Table because of the large amount of computer time consumed in so doing and also because the observed values are very close to those expected, such that few of the differences even approach statistical significance. The assumption of a Poisson distribution will in general provide a rough test of significance. For illustrative purposes, standard deviations have been calculated for one set of data in which the number of overlaps exceeds the number expected for each of the 5 critical distances and these are shown in a footnote to the Table. The variances are approximately equal to the expectations suggesting that, in this case, the assumption of a Poisson distribution is not unreasonable. On this basis, only one of the differences between the observed and expected numbers of overlaps is statistically significant at the conventional 5% level (susceptible period from 1 year to 6 months before onset and infective period onset $\pm$ 1 month with critical distance 4 km) and 2 differences approach statistical significance (same time periods as above with critical distances 0·25 km and 0·5 km). However, such results may well have arisen by chance given the number of significance tests we have performed.

We have also postulated susceptibility *in utero* and infectivity from birth to onset of disease and the results of this analysis are shown in Table IV. The only difference which is statistically significant ($P<0{\cdot}05$) in this Table is that associated with a critical distance of 4 km between pairs of cases.

TABLE IV.—*Observed and Expected Overlapping Pairs of Cases for the Postulated Period of Susceptibility being* in utero *and Period of Infectivity being from Birth to Onset of Disease*

| | | Period of infectivity birth to onset | | |
|---|---|---|---|---|
| Period of susceptibility* | "Critical" distance between cases (km) | No. of overlaps | Expected number | Standard deviation |
| Pregnancy | 0·25 | 6 | 4·87 | 1·70 |
| | 0·50 | 18 | 16·36 | 3·03 |
| | 1·00 | 62 | 61·93 | 5·92 |
| | 2·00 | 224 | 214·95 | 11·81 |
| | 4·00 | 840 | 790·70 | 25·85 |

* The period of study is 1 January 1952 to 31 December 1959.

### DISCUSSION

We have been unable to find additional evidence of clustering of the dates and places of birth as reported by Till *et al.* (1967). Studies of space–time clustering of cases of leukaemia, using methods similar to that proposed by Knox, are now numerous and there is probably little to be gained from further studies of this kind.

We have suggested a generalization of the method of Knox which allows for a possibly long and variable latent period. This new method is likely to be of particular value in studying childhood tumours for which induction of the disease might be postulated to take place around the time of birth, followed by a variable latent period until clinical onset. In principle, the technique may be applied to adult tumours also, but for these there is usually no obvious time point about which to define the period of susceptibility. We may, for example, assume that a patient is susceptible from 4 to 5 years before onset, but the assumption of a constant latent period for all patients is unlikely to be correct (as for example, is illustrated by the studies on radiation leukaemogenesis), and there is no obvious way of assigning individual latent periods to patients. This, of course, does not invalidate the statistical test but it is likely to make it a very weak test. We have suggested elsewhere that, in such circumstances, a case-control approach rather than the generalization of Knox's method suggested in this paper is probably a more profitable way to look for evidence of contagion (Pike and Smith, 1974; Smith and Pike, 1974).

An important difference between the method we have used and Knox's method is that in some formulations of the test we are, in effect, testing the postulate that an agent, such as a virus, is transmitted from one case to another. Knox's method provides a test of this, but space–time clustering of cases might also be produced by an environmental agent acting in a limited geographical area for a limited period of time. It is possible that a similar environmental agent might induce leukaemia *in utero* in one child and also bring on the clinical onset of leukaemia in another, thus producing evidence of clustering using the generalized Knox test. However, it seems unlikely that an agent would act in this dual way.

A valid criticism of the method proposed by Knox is that the choice of critical time and space distances is largely arbitrary. Thus, the statistical significance that may be assigned to the findings at a particular time and space distance cannot be evaluated in the usual way, as "significant" results are in most cases obtained after extensive searching of various possible combinations of critical time and space distances. The same problem applies to the method we have proposed. For this method we must specify the periods of susceptibility and infectivity and also the patient's "effective" movements during these time periods. In the absence of any direct evidence that leukaemia in man is contagious *at all*, it is difficult to do other than try a variety of possible times and distances and test any that look interesting on a subsequent set of data. The results we have presented do not strongly suggest any such test on other data. With 2 exceptions, none of the differences we have observed are either large or statistically significant. The exceptional cases arise when the period of susceptibility is defined as the period from 1 year to 6 months before onset and the period of infectivity is defined to be a period of 2 months, centred on the date of onset, and when the period of susceptibility is the time spent *in utero* and the period of infectivity is from birth to onset. The excess in the number of "overlaps" occurs when pairs of cases living up to 4 km apart are considered. The differences are only just significant and are not apparent at critical distances of less than 4 km, and may thus merely represent a chance effect rather than a true excess.

However, it might be useful to apply the method we have used to other sets of data as the Greater London area may have been an unsuitable choice of area for studies of contagion. The movement and contacts of people in big cities may not be very well defined by their places of residence (although this is perhaps more likely to be so for children) and studies in rural areas, which have not been subject to extensive population change, are likely to be more fruitful.

A Fortran IV computer programme, which computes the measure of clustering, its expectations and variance, is available from the authors. The programme may also be used to produce a simulation distribution of the measure of clustering; this may be useful when the assumption of a Normal or Poisson distribution seems unreasonable.

In addition to those mentioned in our previous paper, we are very grateful to Dianna Bull for her care in data preparation and for computing assistance. The data were analysed on the ICL 1906A computer at the Oxford University Computer Laboratory and we are grateful to the Director and his staff for their kind assistance. Sir Richard Doll has generously helped and advised us at all stages of this study.

### REFERENCES

BARTON, D. E., DAVID, F. N. & MERRINGTON, M. (1965) A Criterion for Testing Contagion in Time and Space. *Ann. human Genet.*, **29**, 97.

EDERER, F., MYERS, M. H. & MANTEL, N. (1964) A Statistical Problem in Space and Time: Do Leukaemia Cases Come in Clusters? *Biometrics*, **20**, 626.

GLASS, A. G., HILL, J. A. & MILLER, R. W. (1968) Significance of Leukemia Clusters. *J. Pediat.*, **73**, 101.

KNOX, G. (1963) Detection of Low Intensity Epidemicity. *Br. J. prev. soc. Med.*, **17**, 121.

KNOX, G. (1964*a*) Epidemiology of Childhood Leukaemia in Northumberland and Durham. *Br. J. prev. soc. Med.*, **18**, 17.

KNOX, G. (1964*b*) The Detection of Space–Time Interactions. *Appl. Statist.*, **13**, 25.

MANTEL, N. (1967) The Detection of Disease Clustering and a Generalised Regression Approach. *Cancer Res.*, **27**, 209.

PIKE, M. C. & SMITH, P. G. (1968) Disease Clustering: A Generalisation of Knox's Approach to the Detection of Space–Time Interactions. *Biometrics*, **24**, 541.

PIKE, M. C. & SMITH, P. G. (1974) A Case-control Approach to Examine Diseases for Evidence of Contagion, Including Diseases with Long Latent Periods. *Biometrics*, **30**, 263.

PIKE, M. C., WILLIAMS, E. H. & WRIGHT, B. (1967) Burkitt's Tumour in the West Nile District of Uganda, 1961–5. *Br. med. J.*, ii, 395.

SMITH, P. G. & PIKE, M. C. (1974) Case Clustering in Hodgkin's Disease: a Brief Review of the Present Position and Report of Current Work in Oxford. *Cancer Res.*, **34**, 1156.

STEWART, A. & BARBER, R. (1971) Epidemiological Importance of Childhood Cancers. *Br. med. Bull.*, **27**, 64.

TILL, M., HARDISTY, R. M., PIKE, M. C. & DOLL, R. (1967) Childhood Leukaemia in Greater London: a Search for Evidence of Clustering. *Br. med. J.*, iii, 755.